# A Study of Concrete Slab Damage Detection Based on the Electromechanical Impedance Method

**DOI:** 10.3390/s141019897

**Published:** 2014-10-23

**Authors:** Xianyan Hu, Hongping Zhu, Dansheng Wang

**Affiliations:** 1 School of Civil Engineering and Mechanics, Huazhong University of Science and Technology, Wuhan 430074, China; E-Mails: huxianyan728@163.com (X.H.); danshwang@hust.edu.cn (D.W.); 2 Department of Architecture and Materials, Hubei University of Education, Wuhan 430205, China

**Keywords:** PZT, Electromechanical Impedance method, damage index, reinforced concrete slab, damage detection

## Abstract

Piezoelectric lead zirconate titanate (PZT) is being gradually applied into practice as a new intelligent material for structural health monitoring. In order to study the damage detection properties of PZT on concrete slabs, simply supported reinforced concrete slabs with piezoelectric patches attached to their surfaces were chosen as the research objects and the Electromechanical Impedance method (EMI) was adopted for research. Five kinds of damage condition were designed to test the impedance values at different frequency bands. Consistent rules are found by calculation and analysis. Both the root mean square deviation (RMSD) and the correlation coefficient deviation (CCD) damage indices are capable of detecting the structural damage. The newly proposed damage index *R_y_/R_x_* can also predict the changes well. The numerical and experimental studies verify that the Electromechanical Impedance method can accurately predict changes in the amount of damage in reinforced concrete slabs. The damage index changes regularly with the distance of damages to the sensor. This relationship can be used to determine the damage location. The newly proposed damage index *R_y_/R_x_* is accurate in determining the damage location.

## 1. Introduction to the Piezoelectric Impedance Method

With the development of structural health monitoring technology, many intelligent materials are applied to structural health monitoring in civil engineering. In the past decades, the application of PZT has aroused wide interest, and many researchers have tried to make use of PZT in the civil engineering structural health monitoring and put forward many theoretical analysis methods. In particular, the fluctuation method and the mechanical impedance method are popularly used. Liang extracted the inherent frequency of a flexible beam according to the coupling impedance, and used a piezoelectric ceramic as a self-sensing actuator for the first time to conduct experimental damage identification studies on a composite truss structure [1–3]. Many researchers have tried different methods to test and verify the Electromechanical Impedance method (EMI) method. Bhalla deduced the piezoelectric admittance formula. According to experimental tests on reinforced concrete frame structures under the different damage conditions examined, both the conspicuous and inconspicuous damages and their locations could be identified by the RMSD indices. Later on, the EMI technology was introduced in detail, and it was found that this technology could not only be useful in the judging subtle damage in machinery, but also be useful for damage diagnosis of aviation parts and large civil engineering structures [4,5].

Piezoelectric materials are very sensitive to local damages. Many scholars have tested and studied different materials and damage situations to analyze the various damage indices theoretically. Giurgiutiu deduced the impedance formula and effective structure stiffness for a thin slab with PZT. Aviation materials were used to conduct damage tests, and the damage indices were analyzed in the near distance and medium range area. The results showed that judging the damage by a high order power damage index had a better effect [6]. Yee *et al.* proposed to determine the damage using one and two-dimensional equations of Electromechanical Impedance. They compared the differences of RMSD among different numbers of damages, and found that when the frequency band was chosen appropriately, the damage judgment should be effective [7]. Wang embedded the PZT in the concrete. The strength of the concrete test block gradually increased with the age, through monitoring the damage indices of the mean absolute percentage deviation (MAPD) [8]. Na noticed that in concrete structures, the impedance signal of the EMI method would miss a lot of the peak values, which might cause inaccurate assessment of damage changes. Some minor changes would lead to uncertainty in the damage judgment. Under this condition, they made 500 × 300 × 50 mm concrete test blocks, and pasted circular PZT patches on them to collect the data from four frequency bands: 20–50 kHz, 50–80 kHz, 110–140 kHz and 210–240 kHz. It was found that the frequency under 100 kHz could identify the damage better, and the frequency above 100 kHz was not sensitive to damage. Experiments showed that the increased damage index threshold method was feasible in concrete structures [9].

Some researchers have proposed more theories about the mechanical impedance analysis method. It is a challenge to find more effective methods to determine the damage. Wei applied the Timoshenko beam theory in the simulation for a composite beam with cracks. Models were proposed to analyze the crack damage of a multilayer laminated beam [10,11]. A simplified reverberation matrix equation was established, and the beam damages were monitored. Finzi attached more than one piece of PZT to an unconstrained aluminum beam and an aviation aluminum plate. The collection and analysis of data were done by a software system, which could conveniently use the damage indices to make complementary analysis, in cooperation with a wireless communication system [12].

In order to study the performance of PZT damage detection, scholars have considered varied forms of damage, such as holes or notches. Damage indices like RMSD, CCDM and MAPD have been used to test the effectiveness, so as to get rules and practical methods of PZT damage monitoring. Wandowski arranged PZT symmetrically on a 1 m side length aluminum slab in four ways, tested the wave signal, and simulated four kinds of damage. All these ways of arrangement can allow identifying the damage location. The closer the distance to the piezoelectric, the better the testing results. A damage index (DI) was proposed, which can determine different kind of damage effectively [13]. Hamzeloo [14] adopted hollow circular steel tube and aluminum tube specimens, and simulated 10 different kinds of damage. It was found that the damage index not only connected with the damage intensity, but also with the physical properties and the thickness of the specimens [14]. Sepehry *et al.* took PZT as an incentive sensor to test the influence of a radial basis function neural network training method on temperature, by using the damage index RMSD and CC index [15]. Yang has done a series of studies on the application of piezoelectric material in structure health monitoring, in which a reusable embedded PZT was designed to monitor concrete specimens. It was found that a thicker PZT was more sensitive to the signal changes, and the impedance values of the PZT under different constraint conditions and different hydration status were analyzed [16–19]. Although the RMSD index had been adopted by many scholars to judge the damage, it was very difficult to locate precisely. Therefore a new method was proposed, according to which more than one piece of PZT should be attached to the tested objects, and the objects would be monitored in a larger range of frequency (30–400 kHz). The damage should be determined through analyzing the RMSD indices in different frequency bands, and the damage location could then be accurately predicted [20]. The mechanical admittance formula of a two dimensional model was deduced, which was verified by the cantilever aluminum slab [21]. Wang *et al.* calculated the damage indices based on the EMI method and studied the damage location of a beam. The process was simulated by a numerical method, and the results were in good agreement with the experimental results [22].

At present, PZT material has been applied in a large number of fields, due to its small size, low cost, convenient operation, and it can be measured in high frequency range and it is sensitive to local damages. The PZT is used for the damage identification of a reinforced concrete block in this paper. Reinforced concrete blocks were made and they were simply supported with PZT attached to the surface. Firstly a traditional damage index was applied to the analysis, then a new damage index was proposed to carry out studies on damage quantification and damage location, and its accuracy was evaluated.

## 2. PZT-Structure Coupled Model Theory

**Figure 1.**
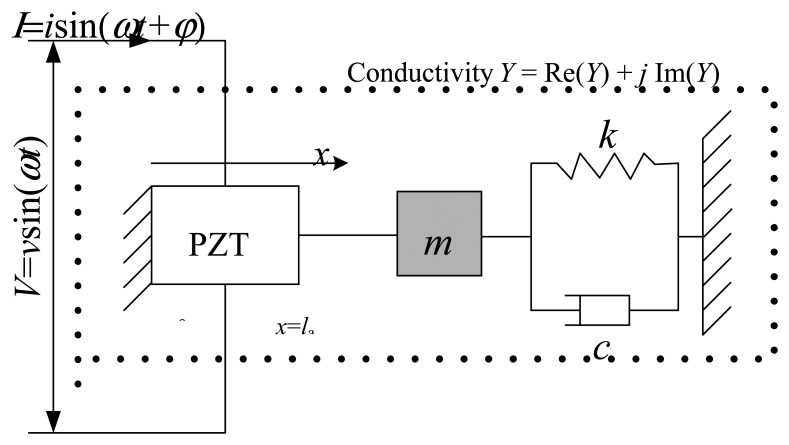
1D model of PZT-structure coupled interactions.

Intelligent structures with piezoelectric patches are widely used in precise positioning control of structures, active control of vibration and repair of cracks. Many researchers have adopted static method and vibration finite element methods to conduct structure vibration analysis. Liang *et al.* thought impedance model technology was more suitable for reflecting the physical essence of a structural system. Taking advantage of the inverse piezoelectric effect of piezoelectric materials, Liang and his colleagues derived a 1D model of the interaction between PZT and structures (Figure 1).

Under the impact of a simple harmonic alternating voltage *V*, the PZT pasted on the monitored structure surface acts as an element, of which one end was fixed, while the other end was connected to the main structure. The PZT impedance values can be expressed by the coupling relationship between the mechanical impedance of the piezoelectric and the main structure driving point. The piezoelectric equation is written as follows:

(1) $$D_{3}=\bar{\varepsilon_{33}^{T}}E_{3}+d_{31}T_{1}$$

(2) $$S_{1}=\frac{T_{1}}{\bar{Y^{E}}}+d_{31}E_{3}$$

in which, *D*_3_ is the electric displacement over the PZT transducer, *S*_1_ the strain in direction 1, and *T*_1_ is axial stress along PZT; $\bar{Y^{E}}=Y^{E}(1+\eta j)$ is the complex elastic Young's modulus of PZT sensor under a constant electric field; $\bar{\varepsilon_{33}^{T}}=\varepsilon_{33}^{T}(1-\delta j)$ is the dielectric constant of PZT sensor under a constant stress, *η* and *δ* are the mechanical loss factor and dielectric loss factor; *d*_31_ is piezoelectric constant.

According to the dynamic equilibrium of PZT sensor, the one-dimensional vibration expression can be expressed by the following equation:

(3) $$\bar{Y_{11}^{E}}\frac{\partial^{2}u}{\partial x^{2}}=\rho \frac{\partial^{2}u}{\partial t^{2}}$$

The size of PZT is 2*l* × *w* × *h*. By integrating over the entire surface of the PZT sensor, we can get the complex piezoelectric admittance (reciprocal of piezoelectric impedance):

(4) $$\bar{Y}=G+jB=\omega j\frac{wl}{h}\times [(\bar{\varepsilon_{33}^{T}}-d_{31}^{2}\bar{Y^{E}})+(\frac{Z_{a}}{Z+Z_{a}})\cdot d_{31}^{2}\cdot \bar{Y^{E}}\cdot (\frac{\tan\kappa l}{\kappa l})]$$

*Z_a_* is the mechanical impedance of PZT under short-circuited condition; *κ* is the number of waves which is related to angular frequency *ω*, density *ρ* and Young's module of an external excitation, and can be described as $\kappa =\omega \sqrt{\rho /\bar{Y^{E}}}$.

If there are damages in structures, structural parameters such as mass M, stiffness K and damping C will likely change. In other words, the mechanical impedance of the structure will change. However, all the parameters of the PZT remain unchanged, so the mechanical impedance of the monitored structure is the only factor that will affect the change of impedance (or admittance). Therefore, any detected change of the impedance signals can be attributed to the damage to the integrity of the structure.

## 3. Damage Index

For health monitoring of structures, an important damage index is indicated by the real part of the PZT sensor's impedance, the changes of which can be easily distinguished and quantified statistically. In structure health monitoring, root mean square index (RMSD) is usually applied to identify damages:

(5) $$\text{RMSD}=\sqrt{\frac{\overset{N}{\underset{i=1}{\text{∑}}}{\left(y_{i}-x_{i}\right)}^{2}}{\overset{N}{\underset{i=1}{\text{∑}}}{\left(x_{i}\right)}^{2}}}$$

*x_i_* and *y_i_* are the impedance measured before and after damage.

Tseng and Naidu took the CC index as damage index [23]. The CC index equals the covariance of two measured data divided by their standard deviation:

(6) $$CC=\frac{\text{Cov}(x,y)}{\sigma_{x}\cdot \sigma_{y}}$$

(7) $$Cov=\frac{1}{N}\sum_{i=1}^{N}\left(x_{i}-\bar{x})(y_{i}-\bar{y}\right)$$

in which, *σ_x_* and *σ_y_* are standard deviation of *x* and *y*, *x̄* and *ȳ* are the mean values of *x* and *y*.

At the same time, CCD and the high power of CCD are used to determine the damage [23,24]:

(8) CCD=1−CC

According to the mathematical statistics theories, a new damage index is put forwarded as:

(9) $$R_{y}/R_{x}=\left|\sqrt{\frac{\sum {\left(y_{i}-\bar{y}\right)}^{2}}{\sum y_{i}^{2}}}-\sqrt{\frac{\sum {\left(x_{i}-\bar{x}\right)}^{2}}{\sum x_{i}^{2}}}\right|/\sqrt{\frac{\sum {\left(x_{i}-\bar{x}\right)}^{2}}{\sum x_{i}^{2}}}$$

where *x_i_* and *y_i_* are the impedance values getting from test before and after the damage, *x̄* and *ȳ* are the average values of *x* and *y*.

## 4. Design of the Test

**Figure 2.**
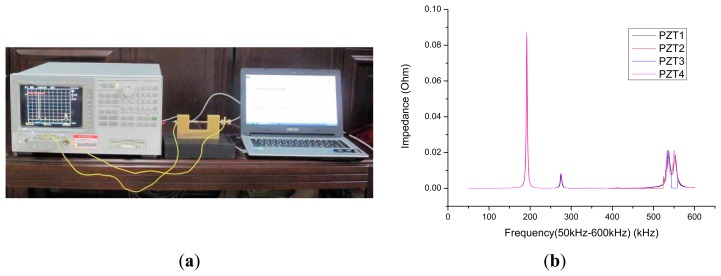
(**a**) Test equipment (**b**) Impedance curve of the bare PZT.

A PIC151piezoelectric ceramic produced by PI Company (Lederhose, Thuringia, Germany) was adopted in the test. The size was 10 × 10 × 0.5 mm, the impedance analyzer was an Agilent 4294A (Agilent Technologies, Santa Clara, CA, USA), the temperature was controlled at 20 ± 2 °C, and impedance signal test was carried out on the bare PZT, as shown in Figure 2.

**Figure 3.**
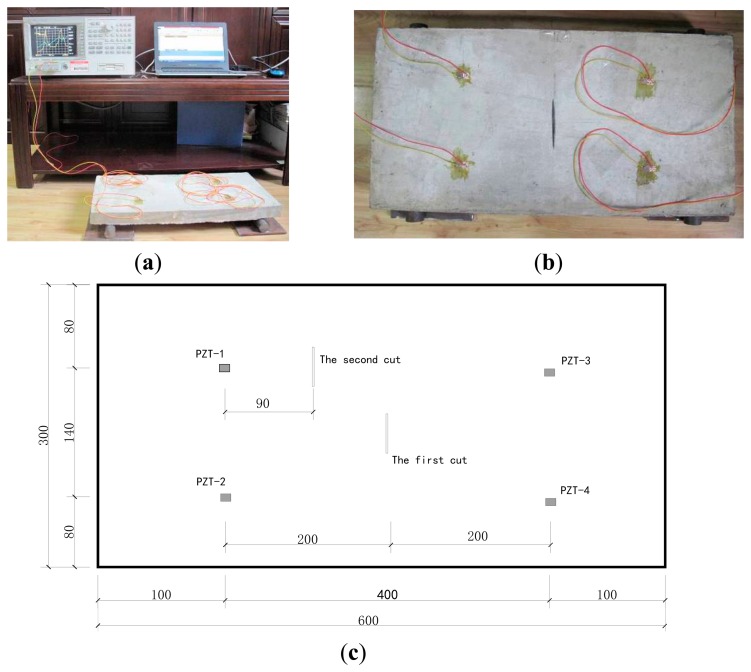
(**a**)Test equipment (**b**) First track of damage in reinforced concrete slab; (**c**) Plan of concrete slab.

In the test, the force of a one-way simply supported slab was simulated. The size of reinforced concrete slab was 600 mm × 300 mm × 40 mm, the concrete grade was C20, and the steel grade was HPB235, and both ends of the slab were simply supported, as shown in Figure 3a. The slab plane was divided into four parts, with each part has one piezoelectric attached to the center of the surface as shown in Figure 3b,c.

First the impedance curve of the undamaged concrete slab was tested, and different damage conditions were set later. Using the cutting machine, we cut the first damage track in the center of the concrete slab, as shown in Figure 3. Afterwards, the cut is enlarged and deepened continually to produce the four conditions. Then the second track of damage was cut at another location on the slab, and all its damage conditions are listed in Table 1. When testing the impedance curve, the frequency bands were divided as follows: 50–600, 50–150, 150–300, 300–450 and 450–600 kHz.

## 5. Discussion

### 5.1. Impedance Curve

**Figure 4.**
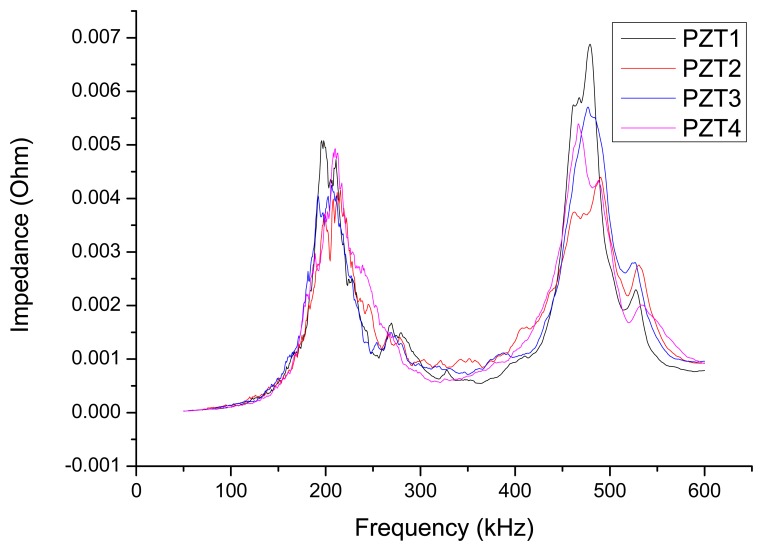
Impedance curve of PZT1-PZT4 (frequency 50–600 kHz).

The real part of the impedance curves of PZT1-PZT4, which is obtained when the concrete slabs are intact, is shown in Figure 4 (the frequency range is 50–600 kHz).

**Figure 5.**
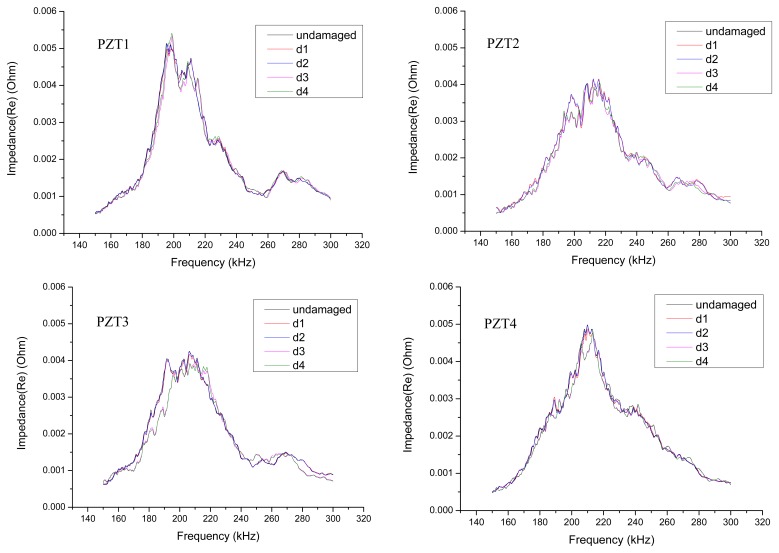
Real part of the undamaged and damaged impedance curves for PZT1–PZT4 (frequency 150–300 kHz).

The test was conducted in four separate frequency bands. Taking the impedance curves of PZT1-PZT4 in the frequency band of 150–300 kHz as an example, the impedance curves of the undamaged condition and the four damage conditions are plotted on the same graph as shown in Figure 5.

**Figure 6.**
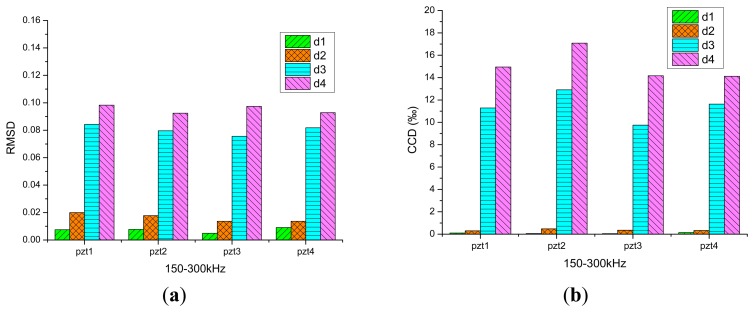

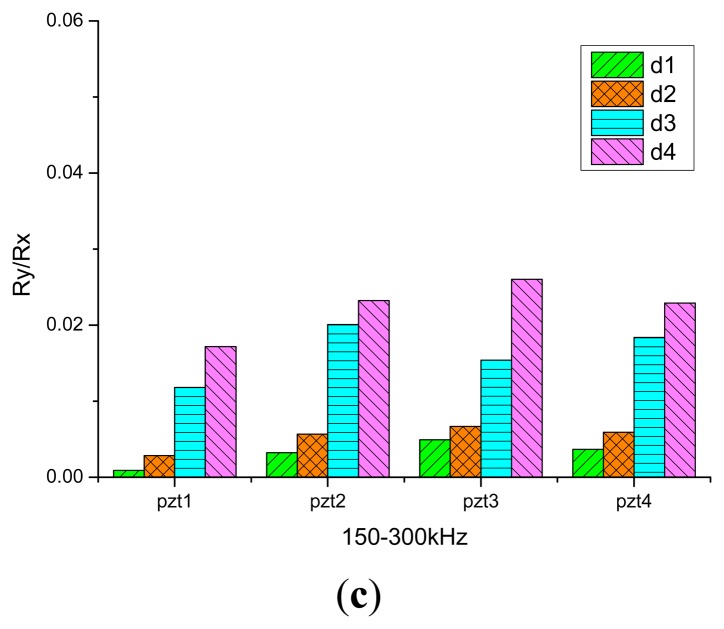
Histogram of the RMSD, CCD and *R_y_*/*R_x_* indices of damage conditions d1–d4.

In the damage analysis of the reinforced concrete slabs, the real part of the impedance curves is used. The frequency range is 150–300 kHz, the RMSD indices and CCD indices of four damage conditions d1, d2, d3, d4 are plotted in histograms in Figure 6a,b. It can be seen from the figure that, along with the increase of the amount of damage, the RMSD indices and CCD indices calculated from the test results of PZT1, PZT2, PZT3 and PZT4 increase, respectively. Because the damage signal of the concrete slabs obtained using the PZT is not sensitive enough, the damage indices of damage conditions d1 and d2 are small. However, when the damage depth of d3 is as deep as 1/2 the thickness of the slab, the damage indices begin to show an obvious growth. Judging from the analysis results, the changes in the amount of damage in reinforced concrete slabs can be monitored by the EMI method.

### 5.2. Damage Index

The new damage index *R_y_/R_x_* is used in the analysis, the frequency band is 150–300 kHz, and the index *R_y_/R_x_* of four damage conditions d1–d4 is plotted in histogram form as shown in Figure 6c. The result shows that along with the increase of the amount of damage, the indices from test results of PZT1, PZT2, PZT3 and PZT4 will increase, respectively. As to condition d3, the damage index also shows a great increase. In consequence, it is reasonable to judge the amount of damage of reinforced concrete slabs with the new index.

### 5.3. The Range of Damage Location

Damage condition d4 is treated as the initial state here, and the second track of damage is d5. The results are analyzed by the RMSD index and *R_y_/R_x_* index. In addition to the quantitative study on reinforced concrete slabs by the EMI method, the study of damage location is also treated in this paper as well.

**Figure 7.**
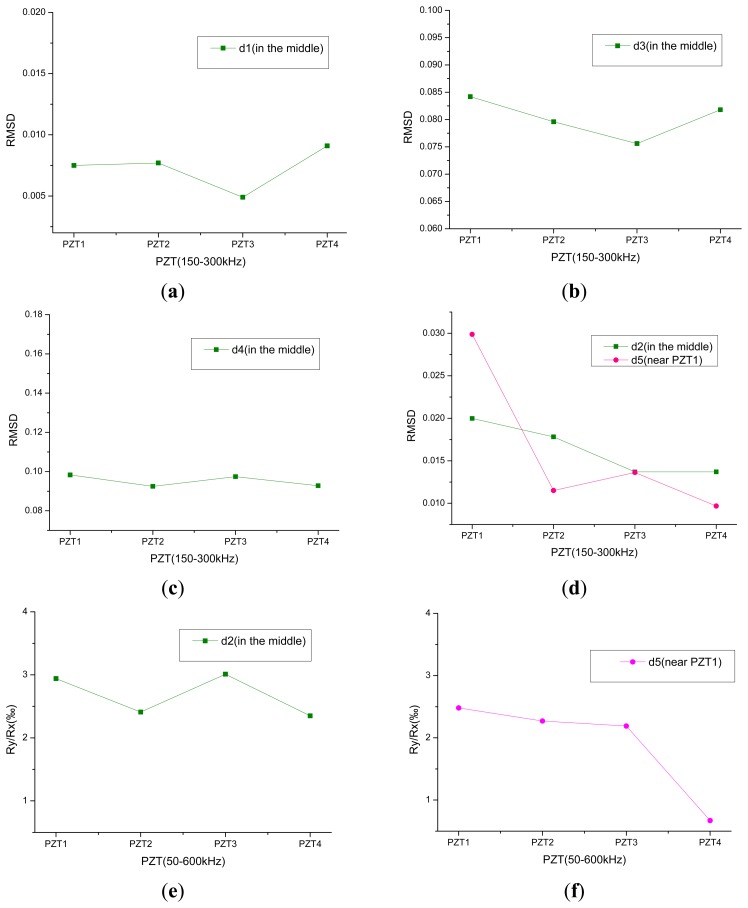
Distribution of damage indices of PZT1-PZT4.

The RMSD index is chosen in the first place, the frequency range is 150–300 kHz, and the indices distribution of damage conditions d1, d3, d4 calculated from PZT1, PZT2, PZT3, PZT4 are plotted in Figure 7a–c. The degrees of the first damage under conditions d2 and the second damage track under conditions d5 are similar, so their distribution is plotted in one figure as shown in Figure 7d. The damages of d1–d4 are in the center of concrete slabs, in the figure green boxes that are basically at the same height, indicating that the four damage indices obtained from the PZT analysis are similar. In Figure 7d, the red dot representing PZT1 is the highest, indicating that there is damage near PZT1.

**Figure 8.**
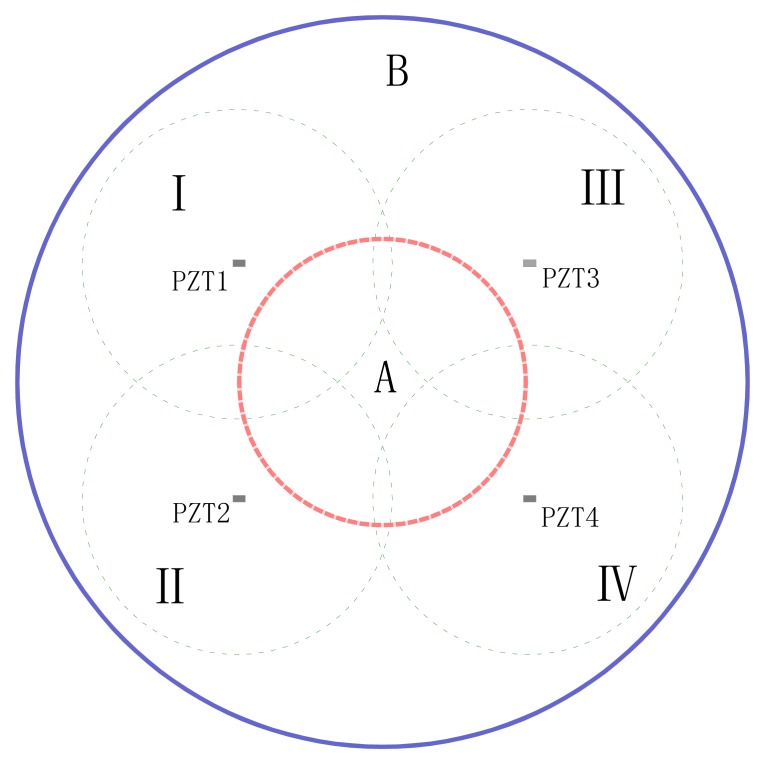
Diagram of the damage areas detected by PZT1-PZT4.

The new index *R_y_/R_x_* is analyzed. The frequency range is 50–600 kHz, and the indices of d2 and d5 from PZT1, PZT2, PZT3, PZT4 are plotted in Figure 7. In Figure 7e, the analysis results obtained by the *R_y_/R_x_* index and RMSD index are the same. Figure 7f shows that the damage is far from PZT4, but in the vicinity of PZT1, PZT2, PZT3, and this area can be roughly located as shown in Figure 8.

It is supposed that the damage areas detected by PZT1–PZT4 are I–IV, as shown in Figure 8. If the damage index values of the four PZT are similar, the damage is near to all four PZT, that is area A; if the difference of one or two indices is bigger, the damage is out of the range of the four PZT, that is area B. In consequence, the possible areas of damage location can be determined by the indices.

Through comprehensive comparison of the two damage indices, it is preliminarily judged that the damages of d1–d4 are near to the four PZT, *i.e.*, they are in area A, and near the center of area A. The damage under condition d5 is near PZT1, around the overlapping area of area I and area A.

## 6. Conclusions

A simply supported reinforced concrete slab is tested with the slab plane divided into four parts. In each of the four divided parts, a piezoelectric is attached to the center of the surface. The EMI method is used to test the impedance values of five kinds of damage condition at different frequency bands: 50–600, 150–300, 300–450 and 450–600 kHz. The test voltage is 1 V and the temperature is controlled at 20 ± 2 °C. The calculation and analysis is conducted using the RMSD and CCD indices. From the calculations it could be seen that the EMI method is not sensitive to the cracks when used to monitor the damage of reinforced concrete material. Along with the increase of the degree of damage, when the depth of the damage was more than 1/2 the thickness of the slab, the damage indices would have a larger growth, and the two different kinds of indices show consistent rules and they could monitor the amount of damage of reinforced concrete slabs well. The newly proposed damage index *R_y_/R_x_* increases as the amount of damage increases. It can monitor the amount of damage of reinforced concrete slabs as well.

Preliminary judgment can be made from the information provided by the four PZT. The damage index changes regularly with the distance between the PZT and the damage location, which is helpful to determine the location of a damaged area. Compared with the RMSD index, the new damage index *R_y_/R_x_* is effective for detecting the damage location, and can give more accurate results.

**Table 1.** Damage conditions set.

**Damage Conditions**		**Length/mm**	**Width/mm**	**Depth/mm**
The first cut	d1	40	4	3
	d2	70	8	5
	d3	80	15	5
	d4	95	25	5

The second cut	d5	70	10	3
